# Regioselective synthesis of pyridines by redox alkylation of pyridine *N*-oxides with malonates

**DOI:** 10.1007/s00706-017-2081-y

**Published:** 2017-11-28

**Authors:** Miran Lemmerer, Christopher J. Teskey, Daniel Kaiser, Nuno Maulide

**Affiliations:** Institute of Organic Chemistry, University of Vienna, Währinger Strasse 38, 1090 Vienna, Austria

**Keywords:** Umpolung, Heterocycles, Nucleophilic additions

## Abstract

**Abstract:**

A regioselective synthesis of pyridines by the addition of malonate anions to pyridine *N*-oxide derivatives, which have been activated by trifluoromethanesulfonic anhydride, is reported. The reaction selectively affords either 2- or 4-substituted pyridines in good yields.

**Graphical abstract:**

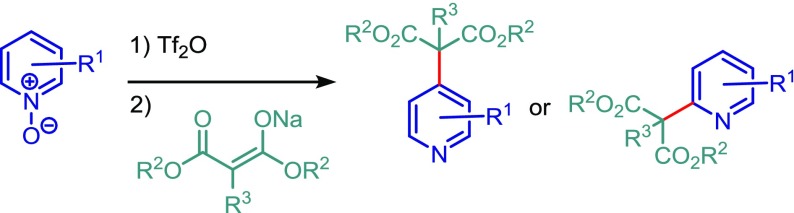

**Electronic supplementary material:**

The online version of this article (10.1007/s00706-017-2081-y) contains supplementary material, which is available to authorized users.

## Introduction

**Fig. 1:**
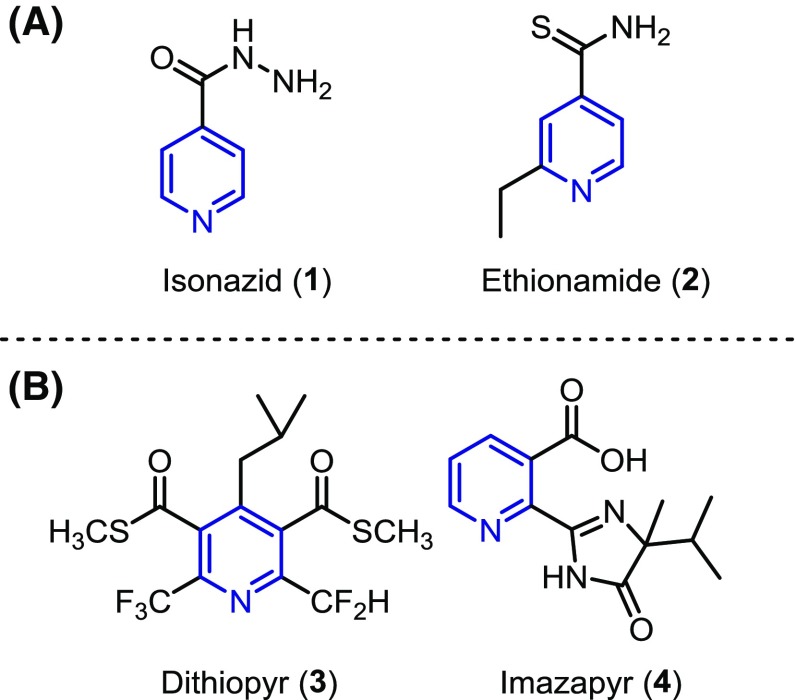
Examples of common **a** drugs and **b** herbicides which contain a pyridine ring

Pyridine is the most common aromatic heterocycle in FDA approved drugs [1]. Significant examples include isoniazid (**1**) and ethionamide (**2**) which are both antibiotics used to treat tuberculosis and are included on the World Health Organizations List of Essential Medicines (Fig. 1a) [2]. A number of herbicides also contain the pyridine motif such as dithiopyr (**3**) [3] and imazapyr (**4**) [4] (Fig. 1b).

As a result of the prevalence of this heterocycle there is a continued interest in the synthesis of densely functionalized examples. Classical approaches to the synthesis of pyridines include the Chichibabin [5] and Hantzsch condensations [6] and the Kröhnke reaction [7]. More modern approaches have been reported including a copper-catalyzed annulation reaction [8] and metal-free cycloaddition reactions [9–11].

Modification or functionalization of existing pyridine structures can be carried out using a variety of strategies. Metal-catalyzed methods range from cross-coupling reactions, such as the Suzuki–Miyaura coupling [12] and iron-catalyzed cross coupling with Grignard reagents [13] to direct C–H functionalization [14]. Minisci reported the addition of carbon-centered radicals to pyridine [15], although this approach is not always completely selective [16]. Another approach to introduce functional groups that avoids the use of metal catalysis is by electrophilic activation of the corresponding *N*-oxide followed by nucleophilic substitution. In 1966, Bauer and Hirsch reported the synthesis of mercaptopicolines via addition of thiols to picoline *N*-oxide which had been activated with phenylsulfonyl chloride [17]. More recently, Johnson et al. have shown how to introduce a protected amine to the 2-position of picoline (Scheme 1A) [18]. Londregan et al. reported that the amide coupling reagent PyBroP can be used to activate pyridine *N*-oxides for the attack of a range of nucleophiles (Scheme 1B) [19].

**Figure:**
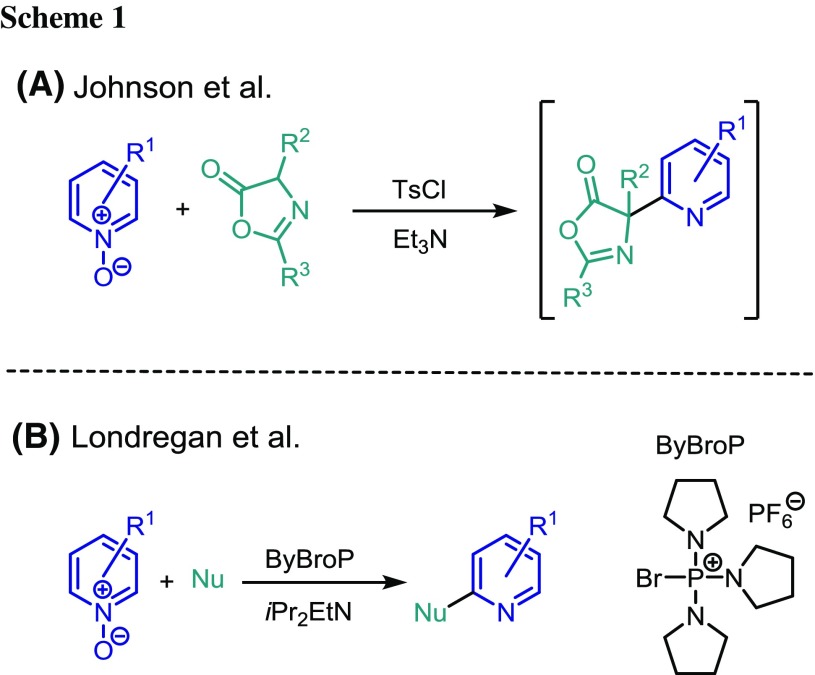

Our group has a long-standing interest in the chemistry of highly reactive intermediates, and in particular, the use of trifluoromethanesulfonic anhydride (triflic anhydride) as an easily handled, commercially available electrophilic activating agent [20–24]. Given this, we decided to investigate its use as an activating agent for pyridine *N*-oxides with malonates as nucleophiles: malonic esters are a versatile handle for the introduction of carboxylic esters or acids [25].

## Results and discussion

We began by treating 2,6-lutidine *N*-oxide with triflic anhydride (Tf_2_O) to form strongly electrophilic intermediate **5**. The addition of a solution of the sodium salt of dibenzyl malonate, generated by the action of sodium hydride on the malonate in THF, resulted in smooth formation of dibenzyl 2-(2,6-dimethylpyridin-4-yl)malonate (**6a**) (Scheme 2).

**Figure:**
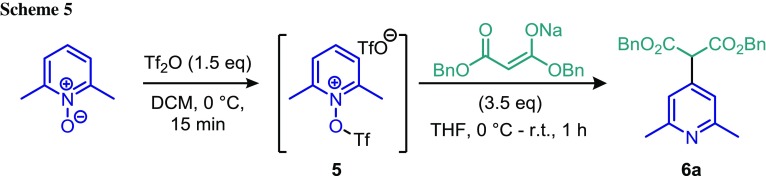

A brief optimization of the reaction conditions yielded a general procedure, whereby the *N*-oxide was treated with 1.5 equivalents of Tf_2_O at 0 °C for 15 min before addition of a THF solution of the malonate anion. This afforded product **6a** in moderate yield of 53%. Using this protocol, we investigated different substitution patterns on the malonate partner (Scheme 3).^1^ We were pleased to find that a fluorine atom could be incorporated giving product **6b** in 61% yield. Gratifyingly, we were able to form quaternary centers (**6c**, **6d**, and **7a**) and both alkene and nitrile functional groups were tolerated on the malonate. We then turned our attention to *N*-oxides that had a pre-existing substituent at the 4-position with the aim to divert functionalization to the 2-position. With diethyl 2-allylmalonate, alkyl (**7b**) and aryl (**7c**) substituents on the *N*-oxide resulted in good yields of the product. However, a nitrile group at the 4-position of the *N*-oxide gave the anticipated product in only poor yield (**7d**). This could be partly due to the reduced nucleophilic character of the *N*-oxide, resulting in a slower reaction with Tf_2_O. Londregan et al. similarly reported a poor yield using his activation procedure. The use of unsubstituted pyridine *N*-oxide yielded a mixture of products alkylated at either the 4- or 2- position (ratio = 1:1.4) in a combined 43% yield.^2^

**Figure:**
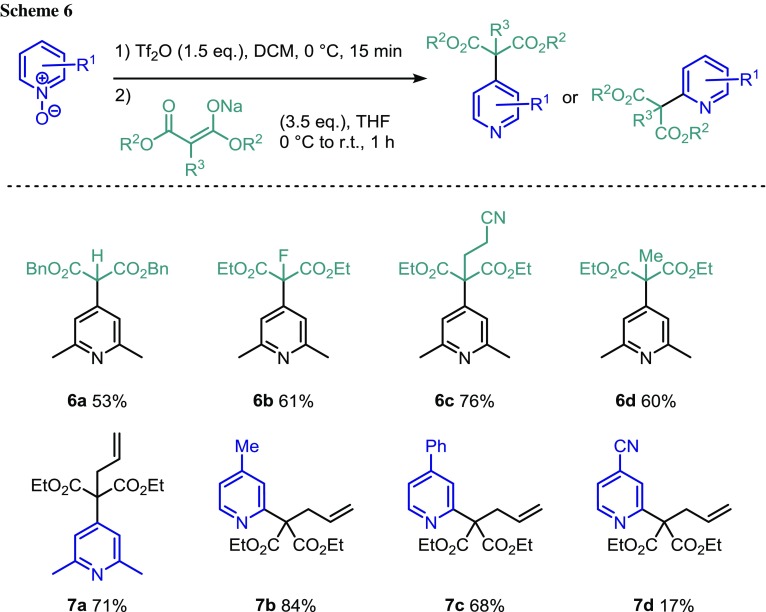

## Conclusion

We have developed a mild and convenient way to functionalize pyridine *N*-oxide derivatives with malonates. This is achieved by activating the corresponding *N*-oxide with Tf_2_O, setting the stage for the nucleophilic addition event. Functional groups including alkenes and nitriles are tolerated on the malonate and this effectively redox-neutral method is amenable to the formation of quaternary centers.

## Experimental

All reagents and anhydrous solvents were used as received from commercial suppliers. Purification was monitored by thin-layer chromatography (TLC) performed on plastic plates coated with Kieselgel F254 with 0.2 mm thickness or GC–MS. Visualization was achieved by ultraviolet light (254 nm) or development with KMnO_4_ solution. Flash column chromatography was performed using silica gel 60 (230–400 mesh, Merck and co.). Near infrared spectra were recorded using a Perkin-Elmer Spectrum 100 FT-IR spectrometer. Mass spectra were obtained using a Finnigan MAT 8200 or (70 eV) or an Agilent 5973 (70 eV) spectrometer, using electrospray ionization (ESI). All ^1^H NMR, ^13^C NMR and ^19^F NMR spectra were recorded on Bruker AV-400 or Bruker AV-600 or Bruker AV-700 in CDCl_3_. Chemical shifts are given in parts per million (*δ*/ppm).

### General procedure

All flasks and stirrer bars were flame dried before use. To the *N*-oxide (0.2 mmol, 1.0 equiv.), dissolved in 2 cm^3^ dichloromethane was added Tf_2_O (0.3 mmol, 1.5 equiv.) at 0 °C. In another flask, a suspension of NaH (0.7 mmol, 3.5 equiv.) in 1 cm^3^ tetrahydrofuran was cooled to 0 °C and the malonate (0.7 mmol, 3.5 equiv.) was added. After 15 min, the malonate solution was added to the activated *N*-oxide solution and the mixture was stirred at room temperature for 1 h. The reaction was quenched with NH_4_Cl solution and the aqueous phase was extracted with dichloromethane. The combined organic layers were washed with brine before being dried over MgSO_4_. The solvents were removed under reduced pressure and the crude product was purified by column chromatography.

#### *Dibenzyl 2*-*(2,6*-*dimethylpyridin*-*4*-*yl)malonate* (6a, C24H23NO4)

The product was prepared according to the general procedure. Purification by column chromatography (EtOAc:heptane = 1:1) yielded the product (41.0 mg, 53%) as a pale yellow solid. ^1^H NMR (400 MHz, CDCl_3_): *δ* = 7.28–7.20 (m, 10H), 6.88 (s, 2H), 5.11 (dd, *J* = 12.0, 18.1 Hz, 4H), 4.56 (s, 1H), 2.43 (s, 6H) ppm; ^13^C NMR (101 MHz, CDCl_3_): *δ* = 167.0, 158.4, 141.5, 135.1, 128.7, 128.7, 128.4, 120.9, 67.9, 57.3, 24.6 ppm; IR: $\bar{\nu }$ = 3064, 3033, 2955, 2922, 1732, 1605, 1569, 1497, 1453, 1375, 1297, 1140 cm^−1^; HRMS (ESI): *m/z* calculated for [M + H]^+^ 390.1700, found 390.1701.

#### *Diethyl 2*-*(2,6*-*dimethylpyridin*-*4*-*yl)*-*2*-*fluoromalonate* (**6b**, C_14_H_18_FNO_4_)

The product was prepared according to the general procedure. Purification by column chromatography (EtOAc:heptane = 1:3) yielded the product (34.3 mg, 61%) as a pale yellow liquid. ^1^H NMR (400 MHz, CDCl_3_): *δ* = 7.19 (s, 2H), 4.33 (q, *J* = 7.1, 4H), 2.56 (s, 6H), 1.32 (t, *J* = 7.1 Hz, 6H) ppm; ^13^C NMR (101 MHz, CDCl_3_): *δ* = 164.9 (d, *J* = 25.0 Hz), 158.3, 142.4 (d, *J* = 22.4 Hz), 116.8 (d, *J* = 9.0 Hz), 93.2 (d, *J* = 202.9 Hz), 63.4, 24.8, 14.0 ppm; ^19^F NMR (659 MHz, CDCl_3_): − 165.2 ppm; IR: $\bar{\nu }$ = 2983, 2927, 1753, 1604, 1569, 1445, 1412, 1369, 1270, 1230, 1174, 1105, 1044, 1010 cm^−1^; HRMS (ESI): *m/z* calculated for [M + H]^+^ 284.1293, found 284.1292.

#### *Diethyl 2*-*(2*-*cyanoethyl)*-*2*-*(2,6*-*dimethylpyridin*-*4*-*yl)malonate* (**6c**, C_17_H_22_N_2_O_4_)

The product was prepared according to the general procedure. Purification by column chromatography (EtOAc:heptane = 1:1) yielded the product (48.4 mg, 76%) as a pink liquid. ^1^H NMR (400 MHz, CDCl_3_): *δ* = 6.90 (s, 2H), 4.32–4.24 (m, 4H), 2.61–2.57 (m, 2H), 2.54 (s, 6H), 2.37–2.33 (m, 2H), 1.28 (t, *J* = 7.1 Hz, 6H) ppm; ^13^C NMR (125 MHz, CDCl_3_): *δ* = 168.8, 158.6, 145.0, 119.0, 118.9, 62.6, 61.3, 32.0, 24.8, 14.0, 13.5 ppm; IR: $\bar{\nu }$ = 2982, 2937, 2249, 1728, 1603, 1564, 1445, 1368, 1254, 1188, 1079, 1016 cm^−1^; HRMS (ESI): *m/z* calculated for [M + H]^+^ 319.1652, found 319.1651.

#### *Diethyl 2*-*(2,6*-*dimethylpyridin*-*4*-*yl)*-*2*-*methylmalonate* (**6d**, C_15_H_21_NO_4_)

The product was prepared according to the general procedure. Purification by column chromatography (EtOAc:heptane = 1:1) yielded the product (33.6 mg, 60%) as a pale yellow liquid. ^1^H NMR (400 MHz, CDCl_3_): *δ* = 6.94 (s, 2H), 4.25 (m, 4H), 2.52 (s, 6H), 1.81 (s, 3H), 1.26 (t, *J* = 7.2 Hz, 6H) ppm; ^13^C NMR (101 MHz, CDCl_3_): *δ* = 170.7, 157.9, 147.8, 119.1, 62.1, 58.6, 24.8, 22.2, 14.1 ppm; IR: $\bar{\nu }$ = 2982, 1728, 1604, 1564, 1447, 1414, 1377, 1253, 1181, 1105, 1017 cm^−1^; HRMS (ESI): *m/z* calculated for [M + H]^+^ 280.1543, found 280.1543.

#### *Diethyl 2*-*allyl*-*2*-*(2,6*-*dimethylpyridin*-*4*-*yl)malonate* (**7a**, C_17_H_23_NO_4_)

The product was prepared according to the general procedure. Purification by column chromatography (EtOAc:heptane = 1:3) yielded the product (43.6 mg, 71%) as a pale yellow liquid. ^1^H NMR (400 MHz, CDCl_3_): *δ* = 7.01 (s, 2H), 5.75–5.64 (m, 1H), 5.08 (m, 1H), 5.04(s, 1H), 4.29–4.16 (m, 4H), 3.00 (d, *J* = 7.1 Hz, 2H), 2.52 (s, 6H), 1.25 (t, *J* = 7.1, 6H) ppm; ^13^C NMR (101 MHz, CDCl_3_): *δ* = 169.5, 157.8, 146.3, 132.5, 119.7, 119.4, 62.4, 62.0, 40.3, 24.8, 14.1 ppm; IR: $\bar{\nu }$ = 2981, 2926, 1729, 1602, 1563, 1443, 1414, 1367, 1295, 1270, 1230, 1196, 1162 cm^−1^; HRMS (ESI): *m/z* calculated for [M + H]^+^ 306.1700, found 306.1703.

#### *Diethyl 2*-*allyl*-*2*-*(4*-*methylpyridin*-*2*-*yl)malonate* (**7b**, C_16_H_21_NO_4_)

The product was prepared according to the general procedure. Purification by column chromatography (EtOAc:heptane = 1:10) yielded the product (48.8 mg, 84%) as a pale yellow liquid. ^1^H NMR (400 MHz, CDCl_3_): *δ* = 8.39 (dd, *J* = 0.5, 5.0 Hz, 1H), 7.56 (app t, *J* = 0.7 Hz, 1H), 7.01–6.99 (m, 1H), 5.82–5.75 (m, 1H), 5.04–4.99 (m, 2H), 4.27–4.20 (m, 4H), 3.12 (d, *J* = 7.2 Hz, 2H), 2.36 (s, 3H), 1.24 (t, *J* = 7.1 Hz, 6H) ppm; ^13^C NMR (101 MHz, CDCl_3_): *δ* = 169.9, 156.6, 148.6, 147.1, 133.6, 124.8, 123.5, 118.6, 65.3, 61.7, 40.4, 21.4, 14.1 ppm; IR: $\bar{\nu }$ = 2980, 2936, 1729, 1601, 1444, 1298, 1195 cm^−1^; HRMS (ESI): *m/z* calculated for [M + H]^+^ 292.1543, found 292.1543.

#### *Diethyl 2*-*allyl*-*2*-*(4*-*phenylpyridin*-*2*-*yl)malonate* (**7c**, C_21_H_23_NO_4_)

The product was prepared according to the general procedure. Purification by column chromatography (EtOAc:heptane = 1:10) yielded the product (48.0 mg, 68%) as a pale yellow liquid. ^1^H NMR (400 MHz, CDCl_3_): *δ* = 8.59 (dd, *J* = 0.7, 5.1 Hz, 1H), 7.88 (dd, *J* = 0.7, 1.7 Hz, 1H), 7.65–7.63 (m, 2H), 7.48–7.41 (m, 4H), 5.86–5.79 (m, 1H), 5.06–5.02 (m, 2H), 4.30–4.24 (m, 4H), 3.17 (d, *J* = 7.1 Hz, 2H), 1.26 (t, *J* = 7.1 Hz, 6H) ppm; ^13^C NMR (125 MHz, CDCl_3_): *δ* = 169.8, 157.2, 149.2, 148.5, 138.6, 133.3, 129.2, 129.1, 127.3, 122.4, 120.7, 119.0, 65.5, 61.8, 40.5, 14.2 ppm; IR: $\bar{\nu }$ = 3062, 2980, 2935, 1729, 1594, 1547, 1467, 1225, 1036 cm^−1^; HRMS (ESI): *m/z* calculated for [M + H]^+^ 354.1700, found 354.1699.

#### *Diethyl 2*-*allyl*-*2*-*(4*-*cyanopyridin*-*2*-*yl)malonate* (**7d**, C_16_H_18_N_2_O_4_)

The product was prepared according to the general procedure. Purification by column chromatography (EtOAc:heptane = 1:10) yielded the product (10.3 mg, 17%) as a pale yellow liquid. ^1^H NMR (400 MHz, CDCl_3_): *δ* = 8.72 (dd, *J* = 0.9, 5.0 Hz, 1H), 7.98 (app t, *J* = 1.3 Hz, 1H), 7.43 (dd, *J* = 1.3, 5.0 Hz, 1H), 5.74–5.63 (m, 1H), 5.04–5.01 (m, 2H), 4.29–4.22 (m, 4H), 3.12 (d, *J* = 7.3 Hz, 2H), 1.25 (t, *J* = 7.1 Hz, 6H) ppm; ^13^C NMR (101 MHz, CDCl_3_): *δ* = 169.0, 158.5, 149.6, 132.4, 126.5, 124.0, 120.5, 119.7, 116.8, 65.3, 62.2, 40.3, 14.1 ppm; IR: $\bar{\nu }$ = 3077, 2981, 2933, 2239, 1730, 1594, 1467, 1299, 1168, 1044 cm^−1^; HRMS (ESI): *m/z* calculated for [M + Na]^+^ 325.1159, found 325.1157.

## Electronic supplementary material

Below is the link to the electronic supplementary material.

Supplementary material 1 (DOCX 973 kb)

## Footnotes

1 The use of reduced amounts of nucleophile led to worse results. For instance, employing two equivalents of the anion of diethyl allyl malonate resulted in a 40% yield (^1^H NMR yield using 1,3,5-trimethoxybenzene as an internal standard).

2 ^1^H NMR yield using 1,3,5-trimethoxybenzene as an internal standard.
