# Supplementary material for: Regioselective synthesis of pyridines by redox alkylation of pyridine N-oxides with malonates
Source: Monatsh Chem. 2017 Nov 28;149(4):715–9. doi: 10.1007/s00706-017-2081-y (PMC5906502; doi:10.1007/s00706-017-2081-y)
Supplement: Supplementary file 1 — Supplementary material 1 (DOCX 973 kb) [file 706_2017_2081_MOESM1_ESM.docx]

**Supporting Information**

**NMR spectra**

Dibenzyl 2-(2,6-dimethylpyridin-4-yl)malonate (**6a**)

^1^H NMR (400 MHz, CDCl_3_)

^13^C NMR (101 MHz, CDCl_3_)

Diethyl 2-(2,6-dimethylpyridin-4-yl)-2-fluoromalonate (**6b**)

^1^H NMR (400 MHz, CDCl_3_)

^13^C NMR (101 MHz, CDCl_3_)

^19^F NMR (659 MHz, CDCl_3_)

Diethyl 2-(2-cyanoethyl)-2-(2,6-dimethylpyridin-4-yl)malonate (**6c**)

^1^H NMR (400 MHz, CDCl_3_)

^13^C NMR (125 MHz, CDCl_3_)

Diethyl 2-(2,6-dimethylpyridin-4-yl)-2-methylmalonate (**6d**)

^1^H NMR (400 MHz, CDCl_3_)

^13^C NMR (101 MHz, CDCl_3_)

Diethyl 2-allyl-2-(2,6-dimethylpyridin-4-yl)malonate (**7a**)

^1^H NMR (400 MHz, CDCl_3_)

^13^C NMR (101 MHz, CDCl_3_)

Diethyl 2-allyl-2-(4-methylpyridin-2-yl)malonate (**7b**)


^1^H NMR (400 MHz, CDCl_3_)

^13^C NMR (101 MHz, CDCl_3_)

Diethyl 2-allyl-2-(4-phenylpyridin-2-yl)malonate (**7c**)

^1^H NMR (400 MHz, CDCl_3_)

^13^C NMR (125 MHz, CDCl_3_)

Diethyl 2-allyl-2-(4-cyanopyridin-2-yl)malonate (**7d**)

^1^H NMR (400 MHz, CDCl_3_)

^13^C NMR (101 MHz, CDCl_3_)
